# Circular RNA hsa_circ_0023404 promotes the proliferation, migration and invasion in endometrial cancer cells through regulating miR-217/MAPK1 axis

**DOI:** 10.1186/s40001-022-00866-x

**Published:** 2022-11-09

**Authors:** Zhuoying Chen, Meixiu Huang, Jiaying You, Yanhua Lin, Qiaoyun Huang, Caiping He

**Affiliations:** Department of Gynaecology, Fujian Medical Universtiy Affiliated Mindong Hospital, Ningde, Fujian 355000 People’s Republic of China

**Keywords:** hsa_circ_0023404, miR-217, MARK1, Circular RNA, Endometrial cancer

## Abstract

**Background:**

Emerging studies indicated that circular RNA hsa_circ_ 0023404 and its target miR-217/MARK1 axis play a critical role in cancer progression such as non-small cell lung cancer and cervical cancer. However, the role of hsa_circ_0023404/miR-217/MARK1 involved in endometrial cancer (EC) was not investigated yet. The aim of this study is to investigate the functions of hsa_circ_0023404 in endometrial cancer (EC) and the potential molecular mechanism.

**Methods:**

We used RT-qPCR and Western blot approach to detect the expressed levels of related genes in EC cell lines. Transfected siRNAs were applied to knockdown the level of related mRNA in cells. Cell proliferation by CCK-8 assay and colony formation assay were applied to detect cell proliferation. Transwell migration and invasion assay was for detecting the migration and invasion of the cells.

**Results:**

RT-qPCR showed that the levels of hsa_circ_0023404 and MARK1 mRNA were upregulated, but mirR-217 was decreased in three endometrial cancer cell lines. Knockdown of hsa_circ_0023404 by siRNA markedly increased the level of miR-217 and reduced the proliferation of the Ishikawa cells. It also inhibited the cell migration and invasion. Anti-miR-217 can reverse the promoted proliferation, migrations and invasion of Ishikawa cells mediated by si-circ_0023404. si-MARK1 restored the inhibited cell proliferation, migration and invasion of the co-transfected Ishikawa cells with si- circ_0023404 and anti-miR-217.

**Conclusion:**

hsa_circ_0023404 exerts a tumor-promoting role in endometrial cancer by regulating miR-217/MARK1 axis. hsa_circ_0023404 inhibit miR-217 as sponge which inhibit endometrial cancer cell growth and metastasis. MARK1 is downstream target of miR217 and upregulated by hsa_circ_ 0023404/miR-217 axis and involved in the endometrial cancer progression.

**Supplementary Information:**

The online version contains supplementary material available at 10.1186/s40001-022-00866-x.

## Introduction

Endometrial cancer (EC) is one of the most common types of gynecological cancer and the fourth most common cancer among women. Morbidity and mortality rates among patients with EC remain high globally [1]. Each year, approximately 140,000 women worldwide develop endometrial cancer and an estimated 40,000 women die of this cancer. Most cases of EC are diagnosed after menopause and the highest incidence rate is around 70 years old. Survival is usually determined by the stage and histology of the disease, and the prognosis of endometrial cancer varies greatly in different stages and histological types. The most common lesions (type I) are typically hormone-sensitive and in low-stage with good prognosis, while type II tumors have a high grade and are prone to relapse even in the early stages [2].

Recent large-scale genomic studies have shown that a large number of non-coding RNAs (such as microRNAs and long non-coding RNAs) are associated with the occurrence of gynecological diseases [3, 4].Circular RNAs (circRNAs) belongs to a new class of non-coding RNAs and are formed by a peculiar pre-mRNA with a covalently closed continuous loop. Due to its structures, circRNA are resistant to degradation by exonuclease activity and more stable than linear RNAs. circRNAs have been implicated in microRNA (miRNA) sequestration, modulation of protein–protein interactions and regulation of mRNA transcription. Among them, the most striking function is acting as a miRNA sponge and regulate the expression of their downstream genes [5, 6]. MicroRNAs (miRNAs or miRs) are a class of non-coding RNA molecules that negatively regulate the translation of messenger (m) RNAs by interacting with complementary sites in the 3' untranslated region (UTR) [7]. Many miRNAs act as tumor regulator genes by directly targeting oncogenes or tumor suppressor genes [8]. CircRNAs were implicated not only involved in cellular physiological functions, but also in various human pathologies including cancer. It was found that circRNAs are aberrantly modulated in human cancer tissues. Furthermore, research is currently focusing on understanding the possible implications of circRNAs in diagnostics, prognosis prediction, and eventually therapeutic intervention in human cancer [3].

CircRNA hsa_circ_0023404 (chr11: 71668272–71671937) is derived from mRNA of ring finger protein 121 (RNF121, NM_018320). Increasing evidence supported that hsa_circ_0023404 play a critical role in cancer progression. For example, it showed that hsa_circ_0023404 can promote the proliferation, migration and invasion of non-small cell lung cancer (NSCLC) by regulating miR-217/ZEB1 axis [9]. Compared with the miR-con group, overexpression of miR-217 reduced the relative luciferase activity of the pGL3-circ_0023404-WT reporter vector in vitro and strongly validated that hsa_circ_0023404 interacted with and sponged miR-217. Other studies demonstrated that hsa_circ_0023404 was involved in cervical cancer by regulating miR-5047 and miR-136/TFCP2 /YAP pathway [10]. Recently, mounting evidence showed that miR‑217 can regulated tumor biology depending on the cell type [11, 12]. It was observed that the WNT, mitogen‑activated protein kinase (MAPK), and PI3K/AKT signaling pathways were important molecular targets of miR-217 in different cancers and contributed to cancer progression [13]. MAPK1 was identified as a novel miR‑217 target and was a key component of RAS/RAF/MAPK pathway which was found activated in about 30% of all human cancer tissues. Activated MAPK1 translocated to the nucleus and catalyzed the phosphorylation of numerous nuclear transcription factors such as ETS (erythroblast transformation specific), ELK-1 (ETS Like-1 protein), c-Fos and activated variety target genes such as ErbB, VEGF, etc., which contributes to the progression of tumors [14, 15]. Inhibition of MAPK1 was shown to block tumor growth and metastasis in prostate cancer [16]. It was found that miR‑217 can suppress tumorigenicity of colorectal cancer targeting MAPK1 [17].

These indicated that hsa_circ_0023404 and its target miR-217/MARK1 axis play a critical role in cancer progression such as non-small cell lung cancer and cervical cancer, but the role of hsa_circ_0023404/miR-217/MARK1 involved in endometrial cancer was not investigated yet. In this study, we investigated the role of hsa_circ_0023404 in promoting endometrial cancer cells associated with miR-217/MAPK1 axis.

## Methods

### Cell culture

Human endometrial endothelial cell (HEEC) and human endometrial cancer cells (Ishikawa, RL95-2 and KLE) were purchased from the American Type Culture Collection (ATCC, USA) or National Infrastructure of Cell Line Resource (Beijing, China). Cells were incubated in DMEM (Gibco, USA) contained 10% fetal bovine serum (FBS; PAN biotech, Germany) and 1% penicillin/streptomycin (Solarbio, China) at 37 °C and 5% CO_2_.

### RT-qPCR

Total RNA was extracted using Neurozol reagent (Macherey–Nagel, Germany) and cDNA was generated using reverse transcription reagent kit (PROMEGA, USA). Real-time PCR was performed using SYBR Green PCR kit (TaKara, China). U6 and GAPDH are internal controls. The qPCR analysis was then performed on an ABI 7500 Real-time PCR System (Applied Biosystems, Thermo Fisher Scientific, USA) according to the instructions supplied by the manufacturer. The relative expression levels of the genes were calculated by comparing to U6 or GAPDH using 2 − ΔΔCT method. The primers were used as follows:

miR-217 FORWARD: CGCGTACTGCATCAGGAACTG;

miR-217 REVERSE: AGTGCAGGGTCCGAGGTATT;

miR-217-5p RT (anti-miR-217) Primer: GTCGTATCCAGTGCAGGGTCCGAGGTATTCGCACTGGATACGACTCCAAT; U6 FORWARD: CTCGCTTCGGCAGCACA;

U6 REVERSE: AACGCTTCACGAATTTGCGT;

circ_0023404 FORWARD: ACCGTGGCCATGAAGCTATG;

circ_0023404REVERSE: GGTCACCATATTGTAGGAGCGT;

GAPDH FORWARD: AGAAGGCTGGGGCTCATTTG;

GAPDH REVERSE: AGGGGCCATCCACAGTCTTC;

MAPK1 FORWARD: CAGTTCTTGACCCCTGGTCC;

MAPK1 REVERSE: GTACATACTGCCGCAGGTCA.

### Cell transfection

si-NC (negative control) sequence: UUCUCCGAACGUGUCACGUTT, si-circRNA (si-hsa_circ_0023404 #1–3; #3 sequence: GGUUCCUGCUAAUCUAUAATT, miR-217 or anti-miR-217 were synthesized by GenePharma (Shanghai, China). They were transfected in Ishikawa cells using Lipofectamine 3000 Reagent (Life Technologies, USA) and then culture in at 37 °C and 5% CO_2_ for 48–72 h.

### Detection of cell proliferation by CCK-8 assay

Ishikawa cells were plated at 2 × 10E3 cells/well in 96-well plates and grown in medium containing 10% FBS for 24 h. After transfection with siRNA, 10 μl of cell count kit-8 (CCK-8, CK04, Dojindo, Japan) was added into each well and cells were incubated for 2 h in a 5% CO_2_ incubator at 37 °C. The absorbance of each well at 450 nm was read in GloMax™ 96 MICROPLATE (Promega, USA).

### Colony formation assay

Ishikawa cells were transfected with siRNA for 48 h and trypsinized and dispensed into 6-well plates with a density of 800 cells/well. When the number of cells in a colony is more than 50, 10% formaldehyde was employed to fix colonies for 10 min and 0.5% crystal violet was adopted to stain colonies for 5 min. Images were photographed and the number of colonies was calculated by ImageJ.

### Transwell migration and invasion assay

For migration assay, transfected Ishikawa cells (1 × 10E5 cells) were suspended in 200 ul serum-free medium and then seeded on the top chamber. Medium contained 10% FBS was added into the lower chamber. After 24 h of incubation, cells on the lower surface of the lower chamber were fixed with 4% PFA and stained with 0.1% crystal. Cells were counted from five randomly selected microscopic fields. For invasion assay, Transwell inserts (Fisher Scientific, USA) were coated with Matrigel (BD, USA). After 24 h incubation, cells on the upper surface of the Transwell membrane were gently removed, and cells on the lower surface of the Transwell membrane were fixed and stained with crystal violet, counted from five randomly selected microscopic fields.

### Western blotting

Cells were collected and lysed with RIPA buffer (Beyotime, China). Equal amount of protein was separated on SDS-PAGE and transferred to PVDF (Millipore, USA). Then, the membranes were incubated with the primary antibodies anti-MARK1 (Cat:125403, Novopro, China) and anti-actin (Sigma, USA). ECL substrates were used to visualize protein bands (Millipore, USA).

### Statistical analysis

All experiments were replicated thrice and all data were expressed as mean ± standard deviation (SD). The software GraphPad 8.0 were used to carry out all statistical analyzes. Student's t-test and one-way ANOVA followed by Bonferroni 's post hoc test were utilized to analyze 2 or multiple groups, respectively. * means *p* < 0.05; ** means *p* < 0.01; *** means *p* < 0.001.

## Results

### Hsa_circ_0023404, miR-217 and MARK1 mRNA expression in endometrial cancer cell lines

**Fig. 1:**
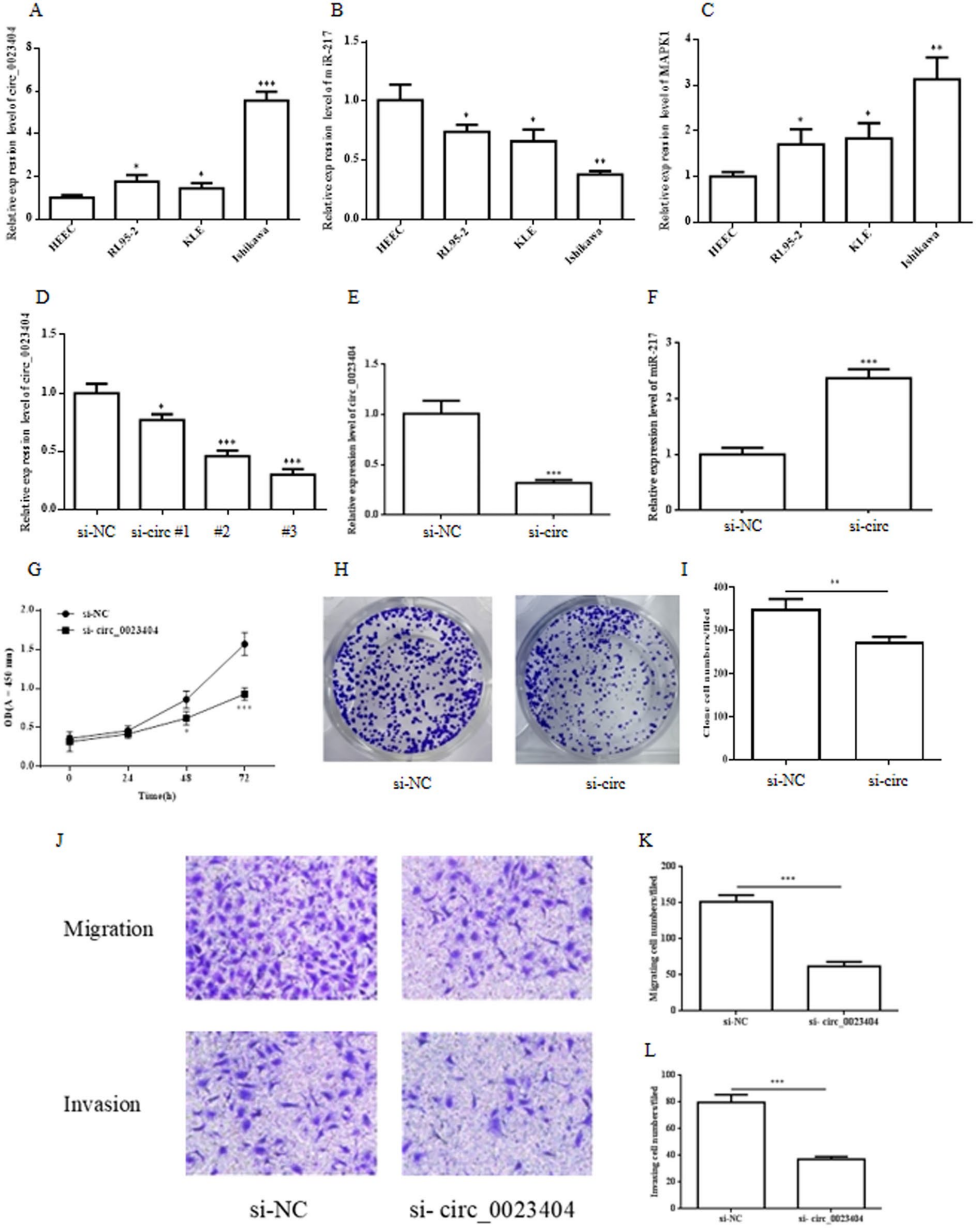
The role of hsa_circ_0023404 knockdown in endometrial cancer cells. The total RNA of HEEC and Ishikawa、RL95-2、 KLE endometrial cancer cell lines were collected and subjected to RT-qPCR. RT-qPCR analysis shows the level of hsa_circ_0023404 (**A**)、miR-217 (**B**) and MARK1 mRNA (**C**) in HEEC and Ishikawa、RL95-2、 KLE endometrial cancer cell lines, respectively. Ishikawa cell was transfected with control si-NC and si-circ_0023404 #1–3 for 48 h and subjected to RT-qPCR analysis. The level of hsa_circ_0023404 was knockdown by si-circ_0023404(si-circ) #1–3 in Ishikawa cell analyzed by RT-qPCR (**D**). The level of hsa_circ_0023404 (**E**) and miR-217 (**F**) was analyzed by RT-qPCR after si-circ #3 transfection. Cell proliferation was evaluated by CCK-8 assay (**G**) and colony formation assay (**H**, **I**) in si-NC or si-circ_0023404 transfected Ishikawa cells. The migration and invasion capacity of Ishikawa cell cells was determined by Transwell migration and invasion assay after transfected with si-NC or si-circ_0023404 (**J**). The statistics of Transwell migration (**K**) and invasion (**L**) assay for Ishikawa cells transfected with si-NC or si-circ_0023404

To examine the role of hsa_circ_0023404 and its target miR-217/MARK1 axis in endometrial cancer cell lines, The RT-qPCR was applied to determine the level of hsa_circ_0023404, miR-217 and MARK1 mRNA in human endometrial endothelial cell (HEEC) and three human endometrial cancer cells (RL95-2, KLE and Ishikawa). It was shown that hsa_circ_0023404 (Fig. 1A) and MARK1 (Fig. 1C) were upregulated in RL95-2, KLE and Ishikawa cell lines compared to HEEC. On the contrary, miR-217 (Fig. 1B) was downregulated in RL95-2, KLE and Ishikawa cell lines compared to HEEC. Among the three cell lines, the results in Ishikawa cell were most strikingly and we employed the Ishikawa cells in further study.

### Knockdown of hsa_circ_0023404 induces miR-217 and inhibits cell proliferation, migration and invasion in endometrial cancer cells

Since the level of hsa_circ_0023404 is upregulated in endometrial cancer cells, we investigate its biological role in endometrial cancer by knockdown of hsa_circ_0023404 with si-circ_0023404 in Ishikawa cells. It showed all three siRNAs targeted to hsa_circ_0023404 significantly reduced mRNA expression of the hsa_circ_002340 in Ishikawa cells compared to control siRNA (si-NC) analyzed by RT-qPCR (Fig. 1D). Among all siRNA, the siRNA#3 had the highest efficiency and was employed for subsequent experiments. We next examined the effect of si-circ_0023404 on miR-217 expression in Ishikawa cells and it showed the si-circ_0023404 #3 reduced the level of circ_ 0023404(Fig. 1E) while the level of miR-217 was upregulated (Fig. 1F). This is consistent with that the circ_0023404 inhibited the miR-217 expression acting as a miRNA sponge. Our data further showed that downregulation of hsa_circ_0023404 markedly decreased the proliferation of the Ishikawa cells detected by CCK-8 assay (Fig. 1G). Down-regulation of hsa_circ_0023404 also markedly decreased the capacity of colony formation of in Ishikawa cells compared to control (Fig. 1H, I). Consistently, knockdown of hsa_circ_0023404 inhibited the cell migration and invasion in Ishikawa cells analyzed by Transwell migrations assay (Fig. 1J, K) and Transwell invasion assay (Fig. 1J, L). These data indicated that hsa_circ_0023404 promoted cell proliferation, migration and invasion in endometrial cancer cells.

### miR-217 inhibits cell proliferation, migration and invasion in endometrial cancer cells

**Fig. 2:**
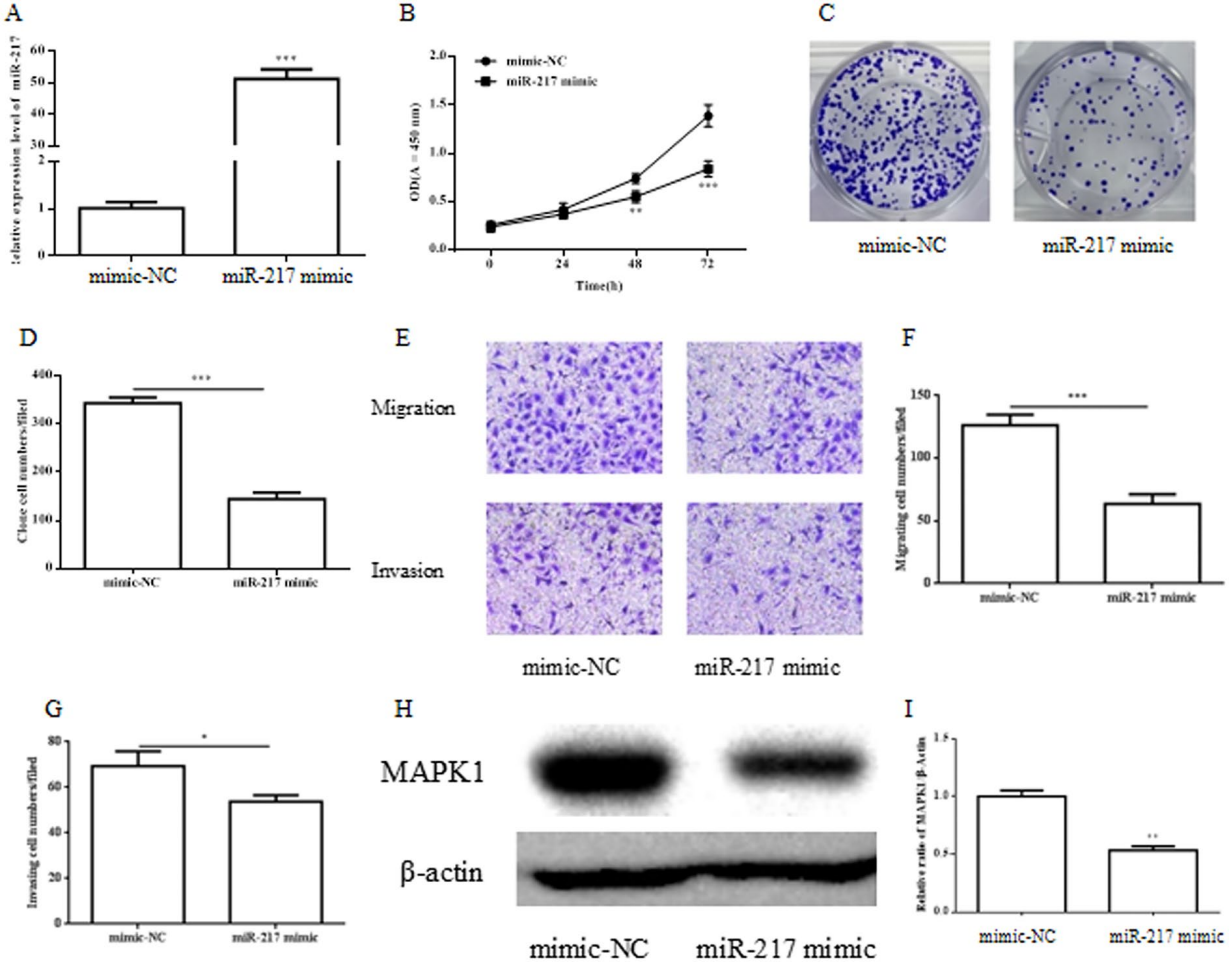
miR-217 inhibits the proliferation of endometrial cancers through by miR-217/MARK1 axis. Ishikawa cell was transfected with control mimic-NC and miR-217 mimic for 48 h and subjected to RT-qPCR analysis to examine the presence of miR-217 (**A**). Cell proliferation was evaluated by CCK-8 assay (**B**) and colony formation assay with statistics (**C**, **D**) after transfection. The migration and invasion capacity of Ishikawa cell cells was determined by Transwell migration and invasion assay after transfection (**E**). The statistics of Transwell migration (**F**) and invasion (**G**) assay were analyzed. Western blot was applied to examine the level of MARK1 protein with an anti-MARK1 antibody (**H**) after transfection with mimic-NC and miR-217 mimic. The statistics of WB were analyzed (**I**)

To investigate the role of miR-217 in endometrial cancer cells, Ishikawa cells were transfected with mimic NC and miR-217 mimic. The expression of transfected miR-217 mimic was confirmed by RT-qPCR (Fig. 2A). CCK-8 assay demonstrated that miR-217 mimic markedly decreased the proliferation of the Ishikawa cells (Fig. 2B). miR-217 mimic also markedly decreased the capacity of colony formation of in Ishikawa cells compared to mimic NC (Fig. 2C, D). In parallel, it showed that miR-217 reduced the cell migration and invasion in Ishikawa cells analyzed by Transwell migrations assay (Fig. 2E, F) and Transwell invasion assay (Fig. 2E, G). MARK1 is the target of miR-217 and WB analysis indicated that the miR-217 mimic transfection decreased the expression of MARK1 protein in Ishikawa cells (Fig. 2H, I). These data indicated that miR-217 played a critical role in inhibiting cell proliferation, migration and invasion in endometrial cancer cells and MARK1 protein is one downstream target of miR-217.

### hsa_circ_0023404 promotes the proliferation, migration and invasion in endometrial cancer cells by sponging miR-217

**Fig. 3:**
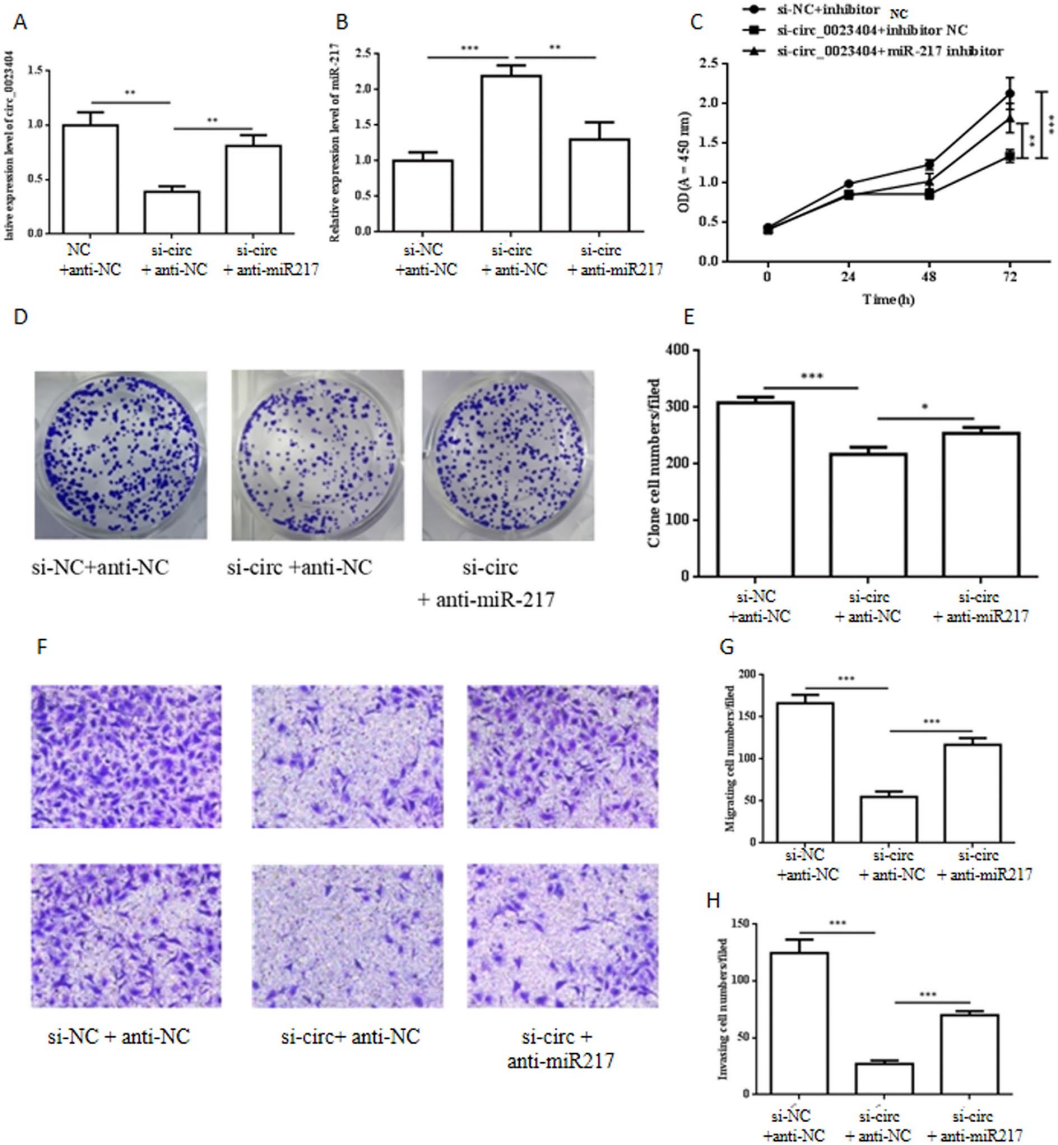
circ_0023404 promotes the proliferation, migration and invasion of endometrial cancers by sponging miR-217. Ishikawa cell was transfected with control si-NC and si-circ_0023404 with anti- NC or anti-miR-217 for 48 h as labeling and subjected to: RT-qPCR analysis to examine the level of circ_0023404 (**A**) and miR-217 (**B**). Cell proliferation was evaluated by CCK-8 assay (**C**) and colony formation assay with statistics (**D**, **E**) after co-transfection. The migration (**F**, top) and invasion (**F**, bottom) capacity of Ishikawa cell cells was determined by Transwell migration and invasion assay after co-transfection. The statistics of Transwell migration (**G**) and invasion (**H**) assay were analyzed

Studies indicated that miR-217 was one sponge target of hsa_circ_0023404 and we examined their interaction by co-transfection with si-circ_0023404 and anti-miR-217 (miR-217 inhibitor). Co-transfection showed that si-circ_0023404 attenuated the expression level of hsa_circ_0023404 while anti-miR-217 increased hsa_circ_0023404 (Fig. 3A); si-circ_0023404 increased the expression level of miR-217 while anti-miR-217 blocked the increased miR-217 (Fig. 3B). CCK-8 assay showed that downregulation of hsa_circ_0023404 decreased the proliferation of the Ishikawa cells but anti-miR-217 reversed the decrease (Fig. 3C, D). Transwell migrations and invasion assay (Fig. 3E, F) also indicated that anti-miR-217 blocked the promoted migrations and invasion of Ishikawa cells mediated by si-circ_0023404. Summary, these data showed that anti-miR-217 can block the promoted proliferation, migrations and invasion of Ishikawa cells by si-circ_0023404, consistent with that hsa_circ_0023404 acts as sponge of miR-217.

### MARK1 is involved in hsa_circ_0023404/miR-217-mediated biological behavior of endometrial cancer

**Fig. 4:**
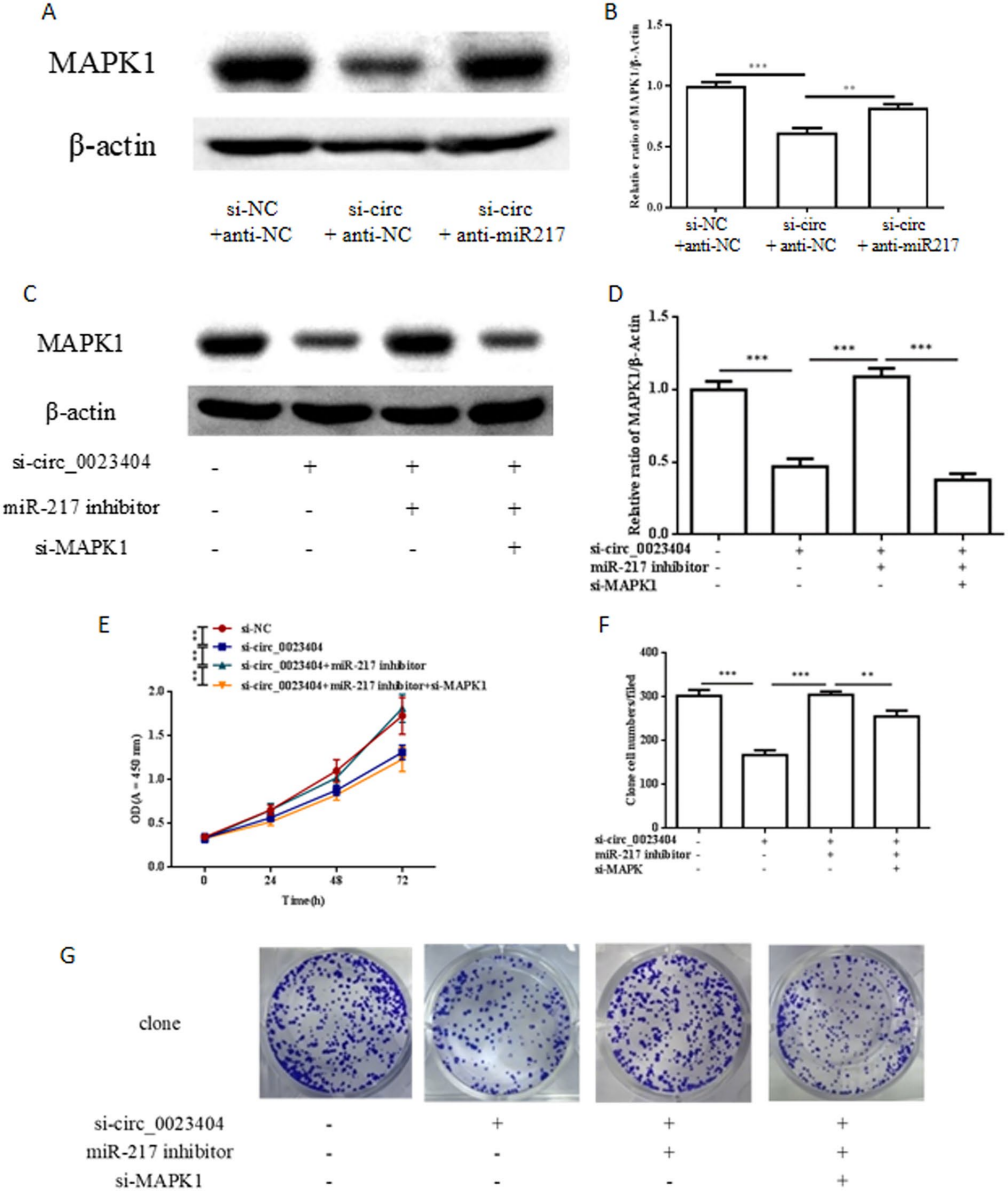
circ_0023404 promotes the proliferation of endometrial cancers through by miR-217/MARK1 axis. Ishikawa cell was transfected with control si-NC or si-circ_0023404 with anti-miR-217 for 48 h and subjected to WB to detect the level of MARK1 protein (**A**) and the statistics was analyzed (**B**). Ishikawa cell co-transfected with si-circ_0023404, anti-miR-217 with si-MARK1 for 48 h and subjected to WB to detect the level of MARK1 protein (**C**) and the statistics was analyzed (**D**). Cell proliferation was evaluated by CCK-8 assay (**E**) and colony formation assay with statistics (**F**, **G**) after co-transfection

**Fig. 5:**
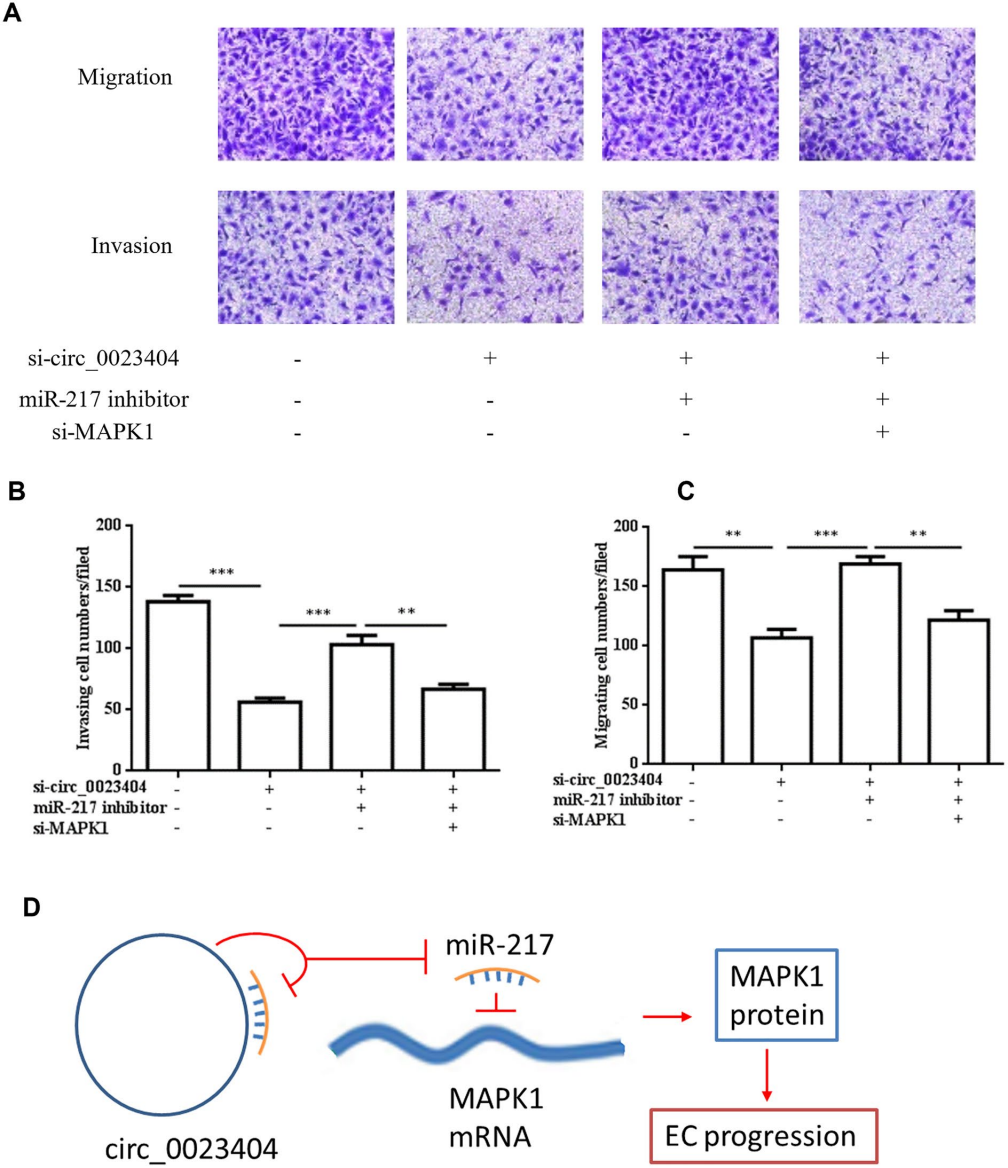
circ_0023404 promotes migration and invasion of endometrial cancers through by miR-217/MARK1 axis. Ishikawa cell co-transfected with si-circ_0023404, anti-miR-217 with si-MARK1 for 48 h and subjected to Transwell migration and invasion assay (**A**). The statistics of Transwell migration (**B**) and invasion (**C**) assay were analyzed. (**D**): schematic diagram of proposed mechanism for circ_0023404-miR-217 MARK1 axis in EC progression. circ_0023404 sponges and inhibits miR-217 which negatively regulates MAPK1 mRNA expression, subsequently leads to increased MAPK1 protein and promotes EC progression

MARK1 is a potential target of miR-217 and our data showed that anti-miR-217 increased the MARK1 protein level which was blocked by co-transfection with si_circ_0023404 and anti-miR-217, supporting that MARK1 is downstream of the hsa_circ_0023404/mirR217 axis (Fig. 4A). Western blot showed that si-MARK1 can downregulate the induced MARK1 by co-transfection with si_circ_0023404 and anti-miR-217. si-MARK1 restored the inhibited cell proliferation of the co-transfected Ishikawa cells with si- circ_0023404 and anti-miR-217 analyzed by CCK8 assay (Fig. 4E) and colony formation assay (Fig. 4F, G). In parallel, it showed that si-MARK1 restored the inhibition in migration (Fig. 5A, B) and invasion (Fig. 5A, C) of the co-transfected Ishikawa cells with si-circ_0023404 and anti-miR-217 analyzed by Transwell migrations and invasion assay. These data supported that the MARK1 is downstream of hsa- circ_0023404/miR-217 axis and MARK1 knockdown by si-MARK1 can block the promotion of cancer biology mediated by si-circ_0023404/miR-217 axis.

## Discussion

Plenty of studies supported that circular RNA hsa_circ_0023404 is associated with tumorigenesis. In this study, we found that hsa_circ_0023404 was upregulated with decreased miR-217 in endometrial cancer cell lines. Knockdown of hsa_circ_0023404 lead to increased miR-217 and inhibited the proliferation and metastasis of endometrial cancer cells. Anti-miR-217 can reverse the imbibition by si-circ_0023404. These data indicated that hsa_circ_0023404 promoted the proliferation, migration and invasion in endometrial cancer cells by sponging miR-217. In further study, knockdown of MARK1 blocked the promotion of cancer biology mediated by si-circ_0023404/miR-217 axis, supporting that MARK1 is the target of miR-217 and involved in circ_0023404/miR-217-mediated endometrial cancer biology.

In human cancer, circRNAs were implicated in the control of oncogenic activities, such as tumor cell proliferation, epithelial–mesenchymal transition, invasion, metastasis and chemoresistance. The most widely described mechanism of action of circRNAs is their ability to act as competing endogenous RNAs (ceRNAs) for miRNAs, lncRNAs and mRNAs, thus impacting along their axis [2, 18, 19]. Several studies revealed that circRNA hsa_circ_0023404 play critical role in tumorigenesis. For example, it enhances cervical cancer metastasis and chemoresistance through VEGFA and autophagy signaling by sponging miR-5047 [10]. hsa_circ_0023404 is also involved in cervical cancer progression through/miR-136/TFCP2/YAP axis. hsa_circ_0023404 promoted TFCP2 expression via inhibiting miR-136, leading to activation of YAP signaling pathway [20]. hsa_circ_0023404 was shown to interact with miR-217/ZEB1 axis to contribute to the growth, migration and invasion of NSCLC cells [9]. This study provided the strong evidence that hsa_circ_0023404 promoted the proliferation, migration and invasion in endometrial cancer cells through regulating miR-217/MARK1 axis.

Dysregulated miRNA expression was involved in malignancies and miRNAs may serve as tumor suppressor or oncogene to participate in human cancer progression. As a miRNA, miR‑217 is closely linked to tumor progression and poor prognosis [21, 22]. Previous studies have reported that miR‑217 bound to its target mRNA to inhibit the formation and progression of tumors, including gastric cancer [22]. Bioinformatics identified MARK1 protein is the target of miR‑217 in cancer cells. There are two binding sequences for miR‑217 in MAPK1 3'UTRs which was confirmed by the luciferase activity assay [23]. Consistently, previous study showed that downregulated MAPK1 by miR-217 facilitated the metastasis and EMT process of HCC cells, indicating that miR-217 suppressed HCC via negatively modulating MAPK1 expression[24]. Mutiple evidence demonstrated that miR217-MAPK axis was involved in tumorgenesis. For example, it was uncovered that the apoptosis-inducing potential of miR-217-5p can induced apoptosis via blocking multiple target genes PRKCI, BAG3, ITGAV and MAPK1 in colorectal cancer cells [25]. It also is reported that circMAN2B2 acted as an onco-miRNA in HCC by sponging miR-217 to promote MAPK1 expression [26].

The MAPK pathway is effectively involved in the regulation of cancer cell proliferation, invasion and survival by activating target genes such as transcriptional factor ELK1, C-Fos and the ErbB, VEGF, which contributes to the progression of tumors [14, 15]. Previous studies have confirmed that increased MAPK1 expression could function as tumor promoter in human hepatocellular carcinoma (HCC) [27, 28], ovarian cancer [29] and cervical cancer [30]. It also showed that lncRNA RHPN1-AS1 activated ERK/MAPK pathway and promoted cell proliferation, migration and invasion of endometrial cancer [31]. Another study demonstrated that activation of MAPK and AKT by Type II transmembrane serine proteases 4TMPRSS4 were associated with the progression of endometrial cancer [32]. These data provided the evidence that MAPK can be regulated by non-coding RNA (ncRNA) including lnRNA and cirRNA, etc. In this study, our data showed that MARK1 is downstream target of hsa_circ_0023404/miR-217 axis and involved in the endometrial cancer progression.

With the advancement of RNA sequencing technology and the rapid development of bioinformatics, a large number of circRNAs were discovered widely involved in a variety of cancer-related pathogenesis and drug resistance and in the diagnostic and prognostic biomarker and the therapeutic target in human cancer [33]. The powerful functions and unique properties of circRNAs have made them the focus of scientific and clinical research. Due to the structure of covalently closed continuous loop, circRNAs are relatively stable and exist stably at high levels in body fluids, including plasma, serum, exosomes and urine, etc. Therefore, circRNA potentially service as the liquid biopsy-based novel biomarkers for monitoring the development and progression of cancer including lung cancer [34], endometrial Cancer [35], bladder cancer [36], prostate cancer [37], etc. Downregulated circBNC2 and higher circSETDB1 levels were identified in patients with ovarian cancer [38]. hsa_circ_ 0109046 and hsa_circ_0002577 were found increase in the serum of patients with endometrial cancer [39]. The unique cellular stability and capacity of circRNA to sponge miRNA and protein may place circRNA as a promising vehicle for the delivery of cancer therapeutics [40].

In the current study, we demonstrated the molecular mechanism of hsa_circ_0023404/miR-217/MAPK involved in the endometrial cancer progression. However, there are several limitation. First, it was limit to draw the conclusion completely only dependent in vitro experiments, therefore, we would further carry out in vivo experiments on hsa_circ_0023404/miR-217/MAPK axis involved in the endometrial cancer. Second, the application of hsa_circ_0023404 on liquid biopsy was not perform on patients with endometrial cancer. We will collect the patients to investigate the level of hsa_circ_0023404/miR-217/MAPK in their blood sample and examine their potential as novel biomarker for endometrial cancer.

## Conclusion

In this study, our data demonstrated that hsa_circ_0023404 exerts a tumor-promoting role in endometrial cancer by regulating miR-217/MARK1 axis. The hsa_circ_0023404 act as sponge for and inhibit miR-217 which inhibit endometrial cancer cell growth and metastasis. MARK1 is downstream target of miR217 and the induced MARK1 by hsa_circ_0023404 through miR217 inhibition contribute to the endometrial cancer progression (Fig. 5D). Targeting or knockdown of hsa_circ_0023404 by short hairpin RNA (shRNA) or CRISPR technique would be a potential therapeutic approach for endometrial cancer and will be investigated in the future.

## Supplementary Information

**Additional file 1: Figure S1.** Uncropped Western blot images.

## Data Availability

The datasets generated and/or analyzed during the current study are available from the corresponding author on reasonable request.
