# Supplementary figures and images for: Circular RNA hsa_circ_0023404 promotes the proliferation, migration and invasion in endometrial cancer cells through regulating miR-217/MAPK1 axis
Source: Eur J Med Res. 2022 Nov 9;27:242. doi: 10.1186/s40001-022-00866-x (PMC9647996; doi:10.1186/s40001-022-00866-x)

Uncropped Western blot images

Fig 2H

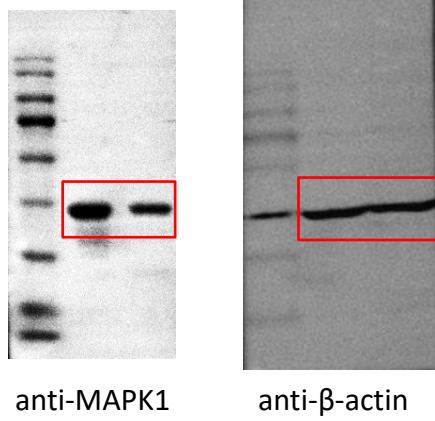

Fig 4A

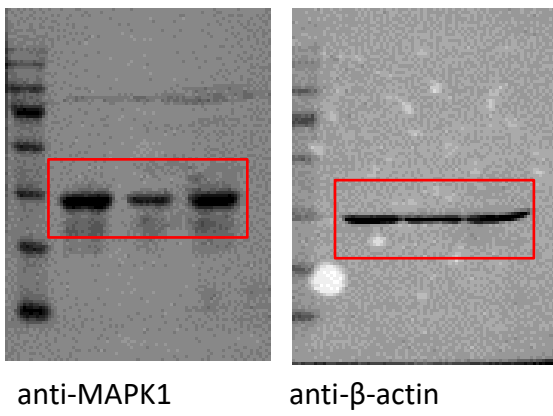

Fig 4C

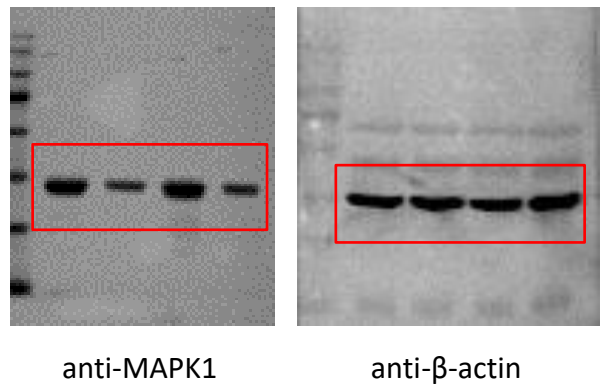

Supplement: Supplementary file 1 — Additional file 1: Figure S1. Uncropped Western blot images. [file 40001_2022_866_MOESM1_ESM.pdf]
